# Development and internal validation of a depression severity prediction model for tinnitus patients based on questionnaire responses and socio-demographics

**DOI:** 10.1038/s41598-020-61593-z

**Published:** 2020-03-13

**Authors:** Uli Niemann, Petra Brueggemann, Benjamin Boecking, Birgit Mazurek, Myra Spiliopoulou

**Affiliations:** 1 Faculty of Computer Science, Otto von Guericke University Magdeburg, Universitätsplatz 2, Magdeburg, 39106 Germany; 2 Tinnitus Center, Charité Universitaetsmedizin Berlin, Charitéplatz 1, Berlin, 10117 Germany

**Keywords:** Depression, Signs and symptoms

## Abstract

Tinnitus is a complex condition that is associated with major psychological and economic impairments – partly through various comorbidities such as depression. Understanding the interaction between tinnitus and depression may thus improve either symptom cluster’s prevention, diagnosis and treatment. In this study, we developed and validated a machine learning model to predict depression severity *after* outpatient therapy (T1) based on variables obtained *before* therapy (T0). 1,490 patients with chronic tinnitus (comorbid major depressive disorder: 52.2%) who completed a 7-day multimodal treatment encompassing tinnitus-specific components, cognitive behavioural therapy, physiotherapy and informational counselling were included. 185 variables were extracted from self-report questionnaires and socio-demographic data acquired at T0. We used 11 classification methods to train models that reliably separate between subclinical and clinical depression at T1 as measured by the general depression questionnaire. To ensure highly predictive and robust classifiers, we tuned algorithm hyperparameters in a 10-fold cross-validation scheme. To reduce model complexity and improve interpretability, we wrapped model training around an incremental feature selection mechanism that retained features that contributed to model prediction. We identified a LASSO model that included all 185 features to yield highest predictive performance (AUC = 0.87 ± 0.04). Through our feature selection wrapper, we identified a LASSO model with good trade-off between predictive performance and interpretability that used only 6 features (AUC = 0.85 ± 0.05). Thus, predictive machine learning models can lead to a better understanding of depression in tinnitus patients, and contribute to the selection of suitable therapeutic strategies and concise and valid questionnaire design for patients with chronic tinnitus with or without comorbid major depressive disorder.

## Introduction

Tinnitus denotes the audiological phantom perception of a sound in the absence of an external source^1^. Tinnitus is a common, yet highly severe worldwide health problem that substantially affects quality of life for millions of people^2,3^. European studies estimate a tinnitus prevalence between 12% and 30%^4^. Besides potential hearing loss^5^, chronic tinnitus is associated with psychological epiphenomena, including anxiety^4,6^, other somatoform disorders^7,8^, insomnia^9^ and, first and foremost, depression^10–12^. Prevalence rates of depression in patients with chronic tinnitus differ considerably, ranging from 14%^13^, to 25.6%^14^ up to 59.3%^15^. In clinical practice, it is often difficult to identify whether a depression symptomatology leads to higher tinnitus distress, or whether a higher tinnitus distress causes a persistent depressive mood. The question of comorbid depression in chronic tinnitus is hence of vital interest – both regarding the conceptualisation and measurement of distress, as well as the identification of possible obstacles to tinnitus-treatment in the face of major depressive disorder. Therefore, it is important to identify the set of variables that should be assessed at baseline to predict clinically relevant depression in tinnitus patients.

At first visit in an outpatient clinic, patients with (chronic) tinnitus usually undergo comprehensive medical and psychological assessments concerning tinnitus distress, loudness and frequency as well as the presence and severity of psychological distress. However, completing multiple lengthy questionnaires can be tedious and cumbersome for patients – often at the expense of accuracy. Hence, it is of interest to identify the most relevant questions clinicians should focus on – thereby reducing the overall amount of questions within a questionnaire. Reducing the burden of questionnaire completion may improve the quality of answers and thus, the assessment’s accuracy.

The traditional approach of extracting the most important questionnaire items requires medical researchers to carefully formulate hypotheses on the relationship between one or more independent variables and the outcome which are statistically validated subsequently. However, due to the increasingly large volume of data which are assessed for each patient, this approach becomes inappropriate, since it is very likely to miss important observations. Hence, to automatically generate new hypotheses in this study we utilize machine learning by building an accurate prediction model by capturing the inherent relationships between its features (the independent variables) and a defined outcome (the dependent variable). The quality and interpretability of such models depend considerably on the selection of relevant features. Ideally, a model is highly accurate while using only a small number of features. Often, there is a trade-off between a complex, highly predictive model and a less complex, yet more *interpretable* and generalisable model that uses fewer features.

While machine learning methods have been extensively used to develop prediction models for depression, e.g. in diabetes patients^16^ and in the general population^17^, we particularly focus on patients with chronic tinnitus.

In this study, we use novel machine learning algorithms (a) to create an accurate model for depression severity after treatment using data extracted from questionnaire answers before treatment, and (b) to minimise the set of predictive features by incrementally removing features on predictive performance. By trading-off high predictive accuracy with low model complexity, our results can help to identify the most important questions that patients may need to answer to accurately assess their depression status.

## Methods

**Figure 1:**
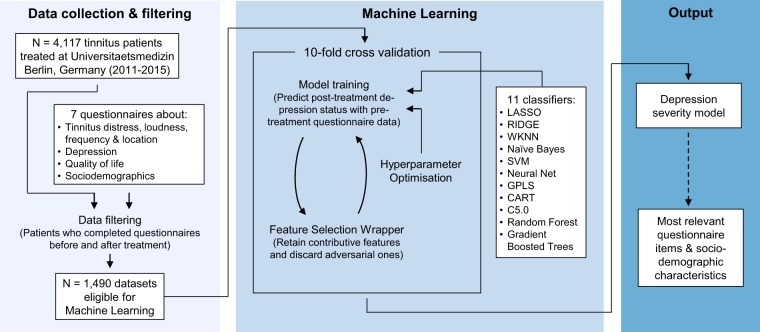
Workflow. We extracted a total of 185 features from answers of 7 questionnaires from 1,490 patients. We trained multiple classification models to predict depression status after outpatient therapy using data collected prior to therapy commencement only. Cross-validation was used for performance evaluation. We embedded model training and evaluation in an incremental feature selection wrapper which retained only features which were identified to be important for the model.

We extracted 185 features from 7 tinnitus-related questionnaires and socio-demographic data for a cohort of 1,490 patients during screening. For these patients, we computed the depression severity after treatment (which lasted 7 days). For prediction of depression severity after treatment, we used the workflow depicted in Fig. 1.

### Features

We used a total of 185 features for data analysis, including single items, sub-scales and total scales from 7 questionnaires: (a) General Depression Scale - long form^18,19^ (“Allgemeine Depressionsskala” - Langform; ADSL), (b) Perceived Stress Questionnaire^20^ (PSQ), (c) Short Form 8 Health Survey^21^ (SF8), (d) German version of the Tinnitus Questionnaire^22^ (TQ), (e) Tinnitus Localisation and Quality^23^ (TLQ), (f) visual analogue scales measuring tinnitus loudness, frequency and distress (TINSKAL), and (g) a sociodemographics questionnaire^24^ (SOZK). Most questionnaire items comprised multiple-choice questions with answers on a Likert scale. The associated ordinal features were handled as numerical features in the analysis. Categorical features, e.g. sex, marital status and graduation, were binarised using one-hot encoding. A brief overview of all features is provided in Supplementary-A.

### Dataset

**Figure 2:**
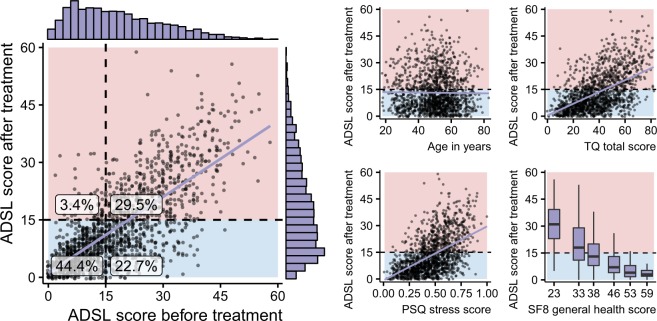
Relationship between depression score after therapy and other features. Graphical representation of the relationship between the ADSL depression score at the end of therapy (y-axis) with other features (x-axis). Higher values on y-axis represent higher depression severity. Background color represents subclinical (blue) or clinical (red) depression status at the end of therapy. Slight jittering was applied to the points to mitigate overplotting. Marginal histograms depict univariate feature distributions.

We used data from a cohort of a total of 4,117 tinnitus patients who had been treated at Tinnitus Center, Charité Universitaetsmedizin Berlin, Germany, between January 2011 and October 2015. All included patients had been suffering from tinnitus for 3 months or longer, were 18 years of age or older and had sufficient knowledge of the German language. Treatment comprised an intensive, multimodal and tinnitus-specific 7-day programme that included informational counselling, detailed ENT as well as psychosomatic and psychological diagnostics, cognitive-behaviour therapy interventions, relaxation exercises, and physiotherapy. Ethical approval was granted by the Charité Universitaetsmedizin Ethics Committee (reference number EA1/115/15) and informed written consent was received from all patients. All methods were performed in accordance with the relevant guidelines and regulations. Prior to the analyses, all data had been anonymised. Patients who did not complete all 7 questionnaires both before and after outpatient therapy were excluded from data analysis. From the remaining 1,502 patients, 12 patients with any missing values were excluded leaving 1,490 datasets included in the analysis. Tinnitus distress was measured by the TQ total score^22^ with a distress-cutoff value of 46^22^ distinguishing between “compensated” (0–46) and “decompensated” (47–84) tinnitus. Table 1 depicts baseline characteristics of all 1,490 included patients before treatment with respect to their tinnitus distress status. The distribution of the defined outcome, the discrete additive depression score (ADSL_adsl_sum) for the patients prior to and after treatment, is shown in Fig. 2. The mean score upon commencing the therapy was 18.2 $\pm$ 11.7 which was significantly larger ($p < 0.001$) than the mean score at the end of the therapy (13.2 $\pm$ 10.7), indicating a positive effect of the multimodal treatment. The target variable “depression status” was created by dichotomising the depression score using a cutoff of 16^19^ distinguishing between “subclinical” (0–15) and “clinical” (16–60) depression. The rate of clinical depression in 755 female patients was 58.6% and significantly larger than the rate of clinical depression in 735 male patients of 45.7% ($p\, <$ 0.001, Chi-square test). The mean patient age was 49.8 years (SD 12.2 years).

**Table 1:** Baseline characteristics of patients before treatment commencement (T0).

		Tinnitus status		
	Total	compensated	decompensated	$p$-value
Number of subjects, n (%)	1490 (100)	1005 (67)	485 (33)	
Age in years	49.8 $\pm$ 12.2	49.3 $\pm$ 12.4	50.8 $\pm$ 11.6	0.023 (TT)
Male sex, n (%)	735 (49)	514 (51)	221 (46)	0.050 (Chi)
Tinnitus duration in years, modus (%)	5 (33)	5 (32)	5 (35)	0.008 (MW)
Number of days until start of an intensive treatment	9.5 $\pm$ 27.0	8.9 $\pm$ 25.1	10.8 $\pm$ 30.5	<0.001 (MW)
TQ total score	38.6 $\pm$ 17.2	29.0 $\pm$ 10.9	58.6 $\pm$ 8.4	<0.001 (TT)
PSQ total score	0.5 $\pm$ 0.2	0.4 $\pm$ 0.2	0.6 $\pm$ 0.2	<0.001 (TT)
SF8 general health score	41.6 $\pm$ 7.1	43.5 $\pm$ 6.4	37.6 $\pm$ 6.6	<0.001 (MW)
ADSL depression score	18.2 $\pm$ 11.7	13.7 $\pm$ 9.2	27.3 $\pm$ 10.9	<0.001 (MW)
Clinical depression, n (%)	777 (52)	362 (36)	415 (86)	<0.001 (Chi)

Baseline characteristics for the patients with compensated tinnitus and patients with decompensated tinnitus, respectively. Continuous variables are expressed as mean $\pm$ standard deviation. Categorical variables are expressed as absolute frequency (percentage). $p$-values were calculated by unpaired two-tailed t-test (TT), Chi-square test (Chi) or two-tailed unpaired Mann-Whitney test (MW). TQ: German version of the Tinnitus Questionnaire^22^; PSQ: Perceived Stress Questionnaire^20^; SF8: Short Form 8 Health Survey^21^; ADSL: General Depression Scale Questionnaire - long form^19^.

### Classification model development

We employed eleven machine learning algorithms for classifier training: LASSO^25^ (lasso), RIDGE^26^ (ridge), weighted k-nearest neighbour classifier^27^ (wknn), Naïve Bayes classifier (nb), support vector machine^28^ (svm), a feed-forward neural network with one single hidden layer^29^ (nnet), generalised partial least squares^30^ (gpls), CART decision tree^31^ (cart), C5.0 decision tree^32^ (c5.0), random forest^33^ (rf) and gradient boosted trees^34^ (gbt). 10-fold stratified cross-validation was used for classifier evaluation. In $k$-fold cross-validation, the data is split into $k$ partitions. Each partition serves once as test set for the model which is trained on the remainder of the partitions. Finally, the $k$ performance results are averaged. A grid search was employed for hyperparameter tuning using area under the ROC curve (AUC) as evaluation measure. A detailed description of all tuned parameter values can be obtained from Supplementary-B.

### Feature selection

We created a novel incremental feature selection wrapper. In particular, we adapted the feature importance score for random forests^33^ and its generalisation to any model type^35^ which is referred to as “model reliance”. The model reliance estimates the difference in the model error after a feature’s values are randomly permuted in the dataset. An estimate of the model reliance for a feature $$f\in F$$ with respect to a model $$\zeta $$, a target vector $$y$$, a dataset $${\bf{X}}$$ and a loss function $$L(y,\zeta ({\bf{X}}))$$ is calculated as follows. First, the model error on the original training data $${{\bf{X}}}_{orig}={\bf{X}}$$ is calculated: $${e}_{orig}=L(y,\zeta ({{\bf{X}}}_{orig}))$$. Secondly, the values of $$f$$ are randomly permuted and the model error on the perturbed dataset $${{\bf{X}}}_{perm}$$ is calculated: $${e}_{perm}=L(y,\zeta ({{\bf{X}}}_{perm}))$$. Finally, the model reliance $$MR(f,\zeta )$$ is calculated as ratio of model error with the permuted feature and model error with the original data: $$MR(f,\zeta )=\frac{{e}_{perm}}{{e}_{orig}}$$. A $$MR$$ value greater than 1 suggests that $$f$$ is important, since randomly permuting its values apparently breaks its relationship with the predicted target. Since feature perturbation involves a degree of uncertainty, $$MR$$ estimates can be improved by repeating the whole procedure $$k$$ times and averaging the $$k$$$$MR$$ scores. In this study, $$MR$$ was calculated as average over 10 runs.

In iteration $i=1$, our incremental feature selection wrapper begins by training an initial model ${m}_{1}$ on the full feature set ${F}_{1}=F$. For each feature, the model reliance $MR(f,{m}_{i})$ is calculated. Features with $MR(f,{m}_{i}) > 1$ are retained for iteration $i+1$ while the remaining features are dropped. This procedure continues until either none of the $MR$ values exceed 1, i.e., $\forall f\in {F}_{i}:MR(f,{m}_{i})\ \le \ 1$, or the feature set in iteration $i$ is identical to the feature set in iteration $i-1$, i.e., ${F}_{i}={F}_{i-1}$.

## Results

### Distribution of responses

More than half (52.2%) of the 1,490 subjects suffered from clinical depression either at start (T0) and end of treatment (T1) (Fig. 2). The average difference in ADSL score between T0 and T1 comprised 5.0 points (SD 8.2). Hence, roughly one fifth of the patients (22.7%) showed symptoms of clinical depression at T0, but not at T1. Nearly half the subjects (44.4%) reported subclinical depression at both time points whereas only a minor fraction of patients (3.4%) reported an increase of depression severity. We found a strong correlation between the ADSL sum score at both time points (Spearman $\rho =0.71$). While we found no correlation between the ADSL score at T1 and patient age ($\rho =-\,0.01$), we identified a moderate correlation between the former and the initial values of TQ total score ($\rho =0.53$), PSQ stress score ($\rho =0.53$) and SF8 general health score ($\rho =-\,0.48$).

### Predictive performance of classification models

The classification models predicted depression status after therapy based on questionnaire answers and social data acquired prior to therapy with high AUC. Table 2 depicts the performance of all classification methods across iterations. The lasso classifier constructed the best overall model (iteration $i=1$, AUC: 0.87 $\pm$ 0.04; mean $\pm$ SD), followed by ridge ($i=1$, AUC: 0.86 $\pm$ 0.04) and gbt ($i=1$, AUC: 0.86 $\pm$ 0.04). The AUCs of each classifier’s best model were similar, ranging from 0.81 (c5.0) to 0.87 (lasso).

**Table 2:** Classification performance.

Classification method											
$i$	lasso	ridge	wknn	nb	svm	gpls	nnet	cart	c5.0	rf	gbt
1	**0.867** (185)	**0.864** (185)	**0.853** (185)	**0.852** (185)	**0.851** (185)	0.838 (185)	0.822 (185)	0.795 (185)	0.795 (185)	0.864 (185)	**0.862** (185)
2	0.856 (89)	0.847 (86)	0.845 (98)	0.849 (70)	0.530 (5)	0.836 (80)	0.807 (117)	0.799 (106)	0.803 (103)	0.864 (109)	0.855 (89)
3	0.857 (50)	0.854 (51)	0.845 (65)	0.829 (38)	0.537 (4)	0.836 (47)	0.809 (87)	0.794 (66)	0.803 (62)	0.866 (99)	0.859 (52)
4	0.856 (24)	0.853 (31)	0.837 (40)	0.832 (26)	0.542 (3)	**0.838** (24)	0.801 (59)	0.799 (45)	0.790 (39)	0.865 (85)	0.858 (38)
5	0.853 (17)	0.853 (21)	0.842 (28)	0.838 (15)	0.562 (2)	0.838 (16)	0.793 (45)	0.811 (34)	0.806 (24)	0.865 (77)	0.855 (24)
6	0.854 (10)	0.851 (15)	0.847 (16)	0.841 (13)	—	0.837 (9)	0.810 (25)	0.817 (28)	0.803 (23)	0.863 (75)	0.856 (16)
7	0.850 (6)	0.854 (11)	0.833 (9)	—	—	0.838 (6)	0.812 (21)	0.822 (24)	0.804 (16)	0.864 (69)	0.854 (14)
8	—	0.854 (9)	0.829 (7)	—	—	—	0.852 (12)	0.822 (23)	0.802 (13)	0.865 (64)	0.853 (11)
9	—	0.854 (8)	0.830 (6)	—	—	—	**0.857** (8)	**0.822** (22)	0.802 (12)	0.865 (59)	—
10	—	0.853 (7)	—	—	—	—	0.842 (4)	—	**0.809** (10)	**0.866** (57)	—
11	—	—	—	—	—	—	—	—	—	0.865 (56)	—
12	—	—	—	—	—	—	—	—	—	0.864 (51)	—
13	—	—	—	—	—	—	—	—	—	0.864 (50)	—
14	—	—	—	—	—	—	—	—	—	0.863 (47)	—

Mean cross-validation AUC for each classifier with best parameter configuration and for each iteration ($i$). The number of features are given in parenthesis. The best run per classifier is highlighted in boldface. All methods induce at least one model with AUC of 0.809 or higher. Empty cells indicate that the feature selection wrapper had already been terminated after a previous iteration.

**Figure 3:**
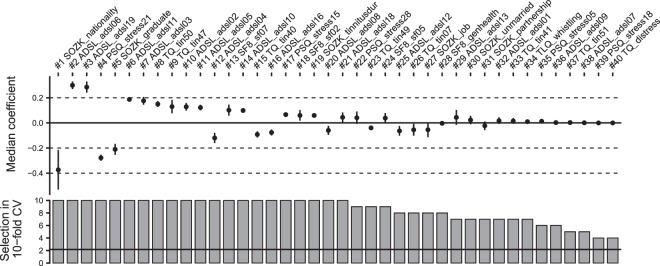
Coefficients and relative inclusion of features in cross-validation of lasso model. Median ($\pm$ median absolute deviation) coefficients (top) and absolute frequency of inclusion of features (bottom) over 10 cross-validation iterations for the best lasso model. From 185 features, the depicted 40 features exhibit a nonzero model coefficient. The average frequency of feature inclusion is represented as horizontal line in the bottom subplot. Line ranges depict MAD (right). TQ: German version of the Tinnitus Questionnaire^22^; PSQ: Perceived Stress Questionnaire^20^; SF8: Short Form 8 Health Survey^21^; ADSL: General Depression Scale Questionnaire - long form^19^; SOZK: sociodemographics questionnaire^24^.

Classification using the best model (lasso, $i=1$) based on a probability threshold of 0.5 resulted in an accuracy of 0.79, a true positive rate (sensitivity) of 0.61, a true negative rate (specificity) of 0.88, a precision of 0.72 and a negative predictive value of 0.82. The final model retained 40 features with nonzero coefficients. Fig. 3 shows the median model coefficient of these features across 10 cross-validation folds. From the ADSL questionnaire, 16 single items were included in the final model. Thus, this questionnaire contributed most to the model prediction. Notably, 5 items from the tinnitus-tailored TQ questionnaire were also included in the model. Further, the model utilised 5 items from the socio-demographics questionnaire (SOZK), including nationality (SOZK_nationality) which appeared to have the highest absolute model coefficient, graduation (SOZK_graduate), tinnitus duration (SOZK_tindur), employment (SOZK_job), marital status (SOZK_unmarried) and partnership status (SOZK_partnership). Table 3 provides a description for each of the 25 features with the largest model coefficient for the lasso model ($i=1$). The complete list of features included in the final model can be consulted in Supplementary-C.

**Table 3:** Top-25 features of lasso model.

Feature	Description	Coefficient
SOZK_nationality	German nationality	−0.370
ADSL_adsl06	“During the past week I felt depressed”.	0.309
ADSL_adsl19	“During the past week I felt that people disliked me”.	0.288
PSQ_stress21	“You enjoy yourself”.	−0.284
SOZK_graduate	Graduation: university	−0.210
ADSL_adsl11	“During the past week my sleep was restless”.	0.196
ADSL_adsl03	“During the past week I felt that I could not shake off the blues even with help from my family or friends”.	0.175
TQ_tin50	Because of the noises I am unable to enjoy the radio or television.	0.151
TQ_tin47	I am a victim of my noises.	0.137
ADSL_adsl02	“During the past week I did not feel like eating; my appetite was poor”.	0.132
ADSL_adsl05	“During the past week I had trouble keeping my mind on what I was doing”.	0.132
SF8_sf07	“During the past 4 weeks, how much have you been bothered by emotional problems (such as feeling anxious, depressed or irritable)?”	0.125
ADSL_adsl10	“During the past week I felt fearful”.	0.107
ADSL_adsl04	“During the past week I felt I was just as good as other people”.	−0.107
TQ_tin40	I am able to forget about the noises when I am doing something interesting.	−0.104
ADSL_adsl16	“During the past week I enjoyed life”.	−0.085
PSQ_stress15	“Your problems seem to be piling up”.	0.081
TQ_tin07	Most of the time the noises are fairly quiet.	−0.069
ADSL_adsl08	“During the past week I felt hopeful about the future”.	−0.064
SF8_sf02	“During the past 4 weeks, how much did physical health problems limit your physical activities (such as walking or climbing stairs)?”	0.059
SOZK_tinnitusdur	“How long have you been suffering from tinnitus (in years)?”	0.058
PSQ_stress28	“You feel loaded down with responsibility”.	0.055
ADSL_adsl18	“During the past week I felt sad”.	0.053
SOZK_job	Job status: currently employed	−0.050
ADSL_adsl13	“During the past week I talked less than usual”.	0.049

Features with highest absolute coefficient in lasso model (iteration $i=1$). TQ: German version of the Tinnitus Questionnaire^22^; PSQ: Perceived Stress Questionnaire^20^; SF8: Short Form 8 Health Survey^21^; ADSL: General Depression Scale Questionnaire - long form^19^; SOZK: sociodemographics questionnaire^24^.

### Stability of classifiers on smaller feature sets

With the exception of svm, all classifiers showed high stability when trained on smaller feature subsets. For example, the difference between lasso on 185 features ($i=1$) and the same on 6 features ($i=7$) was only 0.017 (2% drop). Several classifiers even benefitted from feature selection with respect to predictive performance. For five classifiers (gpls, nnet, cart, c5.0 and rf), the AUC of the model at second or later iteration was larger than the AUC of the first iteration model that used all 185 features. The two decision tree variants cart and c5.0 profited the most from feature selection, since their best performance was reached on the smallest feature subset with a cardinality of 22 and 10, respectively.

### Complexity-interpretability tradeoff

**Figure 4:**
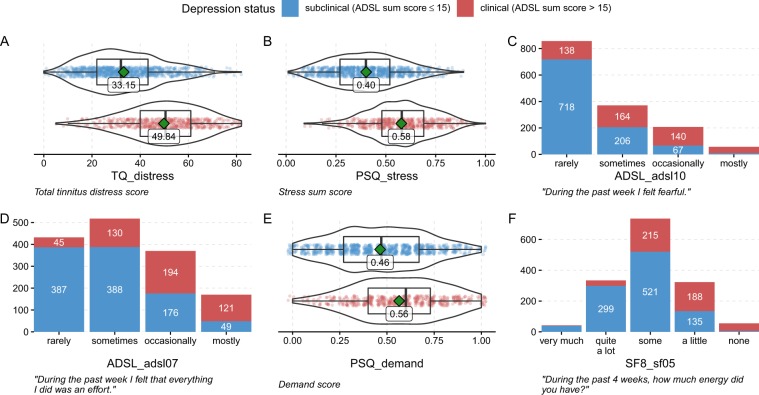
Predictive features. Distribution of features included in the lasso model of iteration $i=7$ for the patients with subclinical and clinical depression. Green squares and labels represent mean of continuous features. ADSL: General Depression Scale Questionnaire - long form^19^; PSQ: Perceived Stress Questionnaire^20^; SF8: Short Form 8 Health Survey^21^; TQ: German version of the Tinnitus Questionnaire^22^.

Our incremental feature selection wrapper reduces the number of features from 185 to 6 without substantial quality loss. The lasso model of iteration $i=7$ provides a reasonable trade-off between a clinically useful predictive quality (AUC: 0.85$\pm$0.05) and a low model complexity (6 features) in comparison with the best overall lasso model (AUC: 0.87 $\pm$ 0.04). Figure 4 depicts a graphical representation of the distribution of these 6 features with respect to depression_status. Patients with clinical depression report a significantly higher mean tinnitus distress score TQ_distress (33.15 $\pm$ 15.2) than patients with subclinical depression (49.8 $\pm$ 15.4) (t-test, $\alpha =0.05$). Analogous, the mean of the stress sum score PSQ_psq_sum (clinical dep.: 0.58 $\pm$ 0.16 vs. subclinical dep.: 0.40 $\pm$ 0.17) and the demand score PSQ_demand (clinical dep.: 0.56 $\pm$ 0.16 vs. subclinical dep.: 0.46 $\pm$ 0.17) were significantly higher for patients with clinical depression. Additionally, three single items were included in the model which showed significant differences with respect to depression_status (Chi-square test, $\alpha =0.05$). For the seventh and tenth question of the ADSL questionnaire (ADSL_adsl07: *“During the past week I felt that everything I did was an effort”*; ADSL_adsl10: *“During the past week I felt fearful”*), the portion of patients with clinical depression ticking answers *“occasionally”* and *“most”* were higher than for *“rarely”* and *“some”*. Accordingly, patients with clinical depression answered the fifth question of the SF8 questionnaire (SF8_sf05: *“During the past 4 weeks, how much energy did you have?”*) rather with *“a little”* or *“none”* instead of *“very much”*, *“quite a lot”* or *“some”*.

## Discussion

Machine learning has been used to create prediction models for depression severity based on structured patient interviews^36,37^. Despite their high predictive performance, we assume that our current models provide a good fit for our sample only, with other subpopulations being yet to be investigated. However, our models are promising and may serve as starting point for timely prediction of depression severity and treatment course with only a small number of questionnaire items.

In agreement with previous studies, the strong association between TQ_distress and depression status indicate a high association between tinnitus-related distress and depressive symptomatology as measured by ADSL^38^. In addition, large model coefficients for PSQ overall score and demand score suggest subjective stress as major contributing factor to depression in tinnitus patients^12^. From a clinical point of view, the inclusion of features from different questionnaires indicates the importance of combining items from several questionnaire types in order to accurately predict depression status. Hence, emotional epiphenomena and other sequelae must be addressed to optimally meet patients’ needs.

A previous study^39^ reported high sensitivity in depression recognition using a questionnaire with only two questions. One of the two questions was “*During the past month, have you often been bothered by feeling down, depressed, or hopeless*.”^39^ which closely resembles the item ADSL_adsl06 (“*During the past week I felt depressed*.”) that exhibited the second-largest absolute coefficient in the best lasso model ($i=1$) in our study.

In general, caution has to be taken when interpreting model coefficients. For example, the lasso model ($i=1$) identified a positive relationship (coefficient: $-0.370$) between non-German citizenship and depression severity (Table 3, Fig. 3). Although ethnical differences in depression were reported in some studies^40,41^, this result rather suggests a higher perceived social stress of predominantly Turkish-born foreign patients, due to higher unemployment rate, larger families, inferior housing, etc. in this demographic group. Further, these results may also be an effect of overfitting, since only 5.0% of the cohort population were non-German citizens. Moreover, the feature had a model reliance score of under 1.0 and consequently was dropped for iteration 2. Although the age feature is included in 8 of the 11 feature sets associated with the best model per classifier, the lack of correlation with the response lets the effect of age on the predicted depression status remains unclear.

With respect to stability of models on a small number of features, it is encouraging that much simpler models are just minorly inferior to the most predictive model. In fact, 5 out of 11 classification algorithms even improved from feature selection, i.e., the AUC at the second or a higher iteration was larger than at the first iteration that uses all features, including the two decision tree variants that reached highest performance on the smallest feature subset, respectively. It is promising that a model (lasso, i = 7) that used only 6 features from 4 questionnaires was only slightly inferior (AUC = 0.850) to the best overall model (AUC = 0.867). For example, neither features on tinnitus localisation and quality, nor sociodemographic features were included in this model. This result could be used to reduce the number of questions or whole questionnaires that the patients have to answer before and after treatment.

The presented study aims at being a first step in providing physicians with guidance for therapy decisions concerning clinical depression in patients with chronic tinnitus. The models could be used to devise a suitable treatment pathway. When applying the models to practice, it is important to notice that they are learned on cross-sectional data, i.e., the model separates between subclinical and clinical depression based on questionnaire answers and socio-demographics before administration of a treatment. Also, the term “clinical depression” refers to how it was modelled in this study, i.e., the depression status after treatment. One has also take into account that the median time difference between start and end of treatment programme was 7 days.

The dataset used for model development might be subject to a selection bias since patients who did not complete all seven questionnaires both during admission *and* after treatment were excluded in the present data analyses. We do not see these data as “missing values” because this might lead to the problematic suggestion of using imputation methods. We cannot use imputation, because (i) a proportion of patients did not complete whole questionnaires (rather than just single items), and (ii) we do not know if data are missing at random. However, given that the number of patients is large, we consider our results as sufficiently robust. In future work, we will investigate potential systematic differences between included and excluded patients. Further, the patient population was obtained from only one German hospital. Hence, the model needs to be externally validated on data from different populations and hospitals.

As another limitation, the incremental feature selection mechanism may miss global optima due to its greedy procedure. At each iteration, only features that are identified to make up for some predictive performance of the classifier are retained and the remaining features are dropped. Once a feature has been eliminated from the feature set, it is not considered at any later iteration. It is possible that the inclusion of a removed feature for classifier training at a later iteration leads to a better model. One possible solution to this problem would be to implement a mechanism which allows for backtracking or revisiting previous iterations. Thus, the $MR$ cutoff value for discarding features could serve as additional tuning parameter. Hence, by testing alternative feature sets at a single iteration, a model with higher predictive performance could be generated.

Motivated by this limitation, future work includes a comparison with other feature selection algorithms. Generally, feature selection algorithms can be roughly divided into embedded methods, filter methods and wrapper methods. Intrinsic methods describe classification methods that internally handle feature selection during model training, e.g., tree- and rule-based classifiers and regularised methods like LASSO. Filter methods are classifier-independent and quantify the *relevance* of a feature before model training by a scoring function. Popular filter approaches are Relief-based methods^42,43^, correlation-based feature selection^44^ and simple statistical scores, e.g., p-value of $t$-test, chi-squared test or Wilcoxon signed-rank test. (Search-based) wrapper methods define a “space” of candidate feature sets. Each candidate feature set is evaluated by a search algorithm which is wrapped around the classifier. To prevent exhaustive search, the search algorithm usually utilises a heuristic to guide the search from the previous best feature set to next best candidate set. Well-known wrapper methods include simple forward/backward selection, recursive feature elimination^45^, simulated annealing^46,47^ and genetic algorithms^48^. The novel feature selection mechanism that is used in this study can be categorised as wrapper method.

Another limitation of this study is the lack of an independent cohort. In future work, the model needs to be externally validated, i.e., tested on data from different centres. Since the use of cross-sectional data currently limits interpretation of the depression status prediction beyond end of therapy, the model needs to be validated with longitudinal data in the future.

## Supplementary information

Supplementary Information.

## Data Availability

Per the Charité Universitaetsmedizin Berlin ethics committee, we cannot make the data public because we do not have the consent of patients to publish their data. Interested researchers can contact the directorate of the Tinnitus Center of Charité Universitaetsmedizin Berlin with data access requests at birgit.mazurek@charite.de.
