# Supplementary material for: Development and internal validation of a depression severity prediction model for tinnitus patients based on questionnaire responses and socio-demographics
Source: Sci Rep. 2020 Mar 13;10:4664. doi: 10.1038/s41598-020-61593-z (PMC7069984; doi:10.1038/s41598-020-61593-z)
Supplement: Supplementary file 1 — Supplementary Information. [file 41598_2020_61593_MOESM1_ESM.pdf]

–Supplementary Material–
Development and internal validation of a depression severity prediction model for tinnitus patients based on questionnaire responses and socio-demographics

Uli Niemann[1*], Petra Brueggemann[2], Benjamin Boecking[2], Birgit Mazurek[2], and Myra Spiliopoulou[1]

[1] *Faculty of Computer Science, Otto-von-Guericke University Magdeburg, Universitätsplatz 2, Magdeburg 39106, Germany*

[2] *Tinnitus Center, Charité Universitaetsmedizin, Charitéplatz 1, Berlin 10117, Germany*

[*] *Corresponding author: uli.niemann@ovgu.de*

Supplementary A: feature overview

Table 1 lists all 185 features that were used for classifier training. These features were extracted from direct question answers or derived scores and measurements from 7 questionnaires: [a] Long Form General Depression Scale [1, 2] (ADSL), [b] Perceived Stress Questionnaire [3] (PSQ), [c] Short Form 8 Health Survey [4] (SF8), [d] German version of the Tinnitus Questionnaire [5] (TQ), [e] Tinnitus Localisation and Quality [6] (TLQ), [f] visual analogue scales measuring tinnitus loudness, frequency and distress (TINSKAL), and [g] a sociodemographics questionnaire [7] (SOZK).

No	Feature	Description
1	ADSL_adsl01	"During the past week I was bothered by things that usually don't bother me."
2	ADSL_adsl02	"During the past week I did not feel like eating; my appetite was poor."
3	ADSL_adsl03	"During the past week I felt that I could not shake off the blues even with help from my family or friends."
4	ADSL_adsl04	"During the past week I felt I was just as good as other people."
5	ADSL_adsl05	"During the past week I had trouble keeping my mind on what I was doing."
6	ADSL_adsl06	"During the past week I felt depressed."
7	ADSL_adsl07	"During the past week I felt that everything I did was an effort."
8	ADSL_adsl08	"During the past week I felt hopeful about the future."
9	ADSL_adsl09	"During the past week I thought my life had been a failure."
10	ADSL_adsl10	"During the past week I felt fearful."
11	ADSL_adsl11	"During the past week my sleep was restless."
12	ADSL_adsl12	"During the past week I was happy."
13	ADSL_adsl13	"During the past week I talked less than usual."
14	ADSL_adsl14	"During the past week I felt lonely."
15	ADSL_adsl15	"During the past week people were unfriendly."
16	ADSL_adsl16	"During the past week I enjoyed life."
17	ADSL_adsl17	"During the past week I had crying spells."

Continued on next page

No	Feature	Description
18	ADSL_adsl18	"During the past week I felt sad."
19	ADSL_adsl19	"During the past week I felt that people disliked me."
20	ADSL_adsl20	"During the past week I could not get going."
21	ADSL_lie	Lie detection score
22	ADSL_timestamp	ADSL: avg. time needed to fill an item (in secs)
23	age	patient age
24	PSQ_demand	demand score
25	PSQ_joy	joy score
26	PSQ_stress	stress sum score
27	PSQ_stress01	"You feel rested."
28	PSQ_stress02	"You feel that too many demands are being made on you."
29	PSQ_stress03	"You are irritable or grouchy."
30	PSQ_stress04	"You have too many things to do."
31	PSQ_stress05	"You feel lonely or isolated."
32	PSQ_stress06	"You find yourself in situations of conflict."
33	PSQ_stress07	"You feel you're doing things you really like."
34	PSQ_stress08	"You feel tired."
35	PSQ_stress09	"You fear you may not manage to attain your goals."
36	PSQ_stress10	"You feel calm."
37	PSQ_stress11	"You have too many decisions to make."
38	PSQ_stress12	"You feel frustrated."
39	PSQ_stress13	"You are full of energy."
40	PSQ_stress14	"You feel tense."
41	PSQ_stress15	"Your problems seem to be piling up."
42	PSQ_stress16	"You feel you're in a hurry."
43	PSQ_stress17	"You feel safe and protected."
44	PSQ_stress18	"You have many worries."
45	PSQ_stress19	"You are under pressure from other people."
46	PSQ_stress20	"You feel discouraged."
47	PSQ_stress21	"You enjoy yourself."
48	PSQ_stress22	"You are afraid for the future."
49	PSQ_stress23	"You feel you're doing things because you have to not because you want to."
50	PSQ_stress24	"You feel criticised or judged."
51	PSQ_stress25	"You are lighthearted."
52	PSQ_stress26	"You feel mentally exhausted."
53	PSQ_stress27	"You have trouble relaxing."
54	PSQ_stress28	"You feel loaded down with responsibility."
55	PSQ_stress29	"You have enough time for yourself."
56	PSQ_stress30	"You feel under pressure from deadlines."
57	PSQ_tension	tension score
58	PSQ_timestamp	PSQ: avg. time needed to fill an item (in secs)
59	PSQ_worries	worries score
60	SF8_bodily_health	Bodily pain score
61	SF8_emotional	Role emotional score
62	SF8_genhealth	General health score
63	SF8_mental	Mental health summary score
64	SF8_mentalhealth	Mental health score
65	SF8_physical	Role physical score
66	SF8_physicalfunc	Physical functioning score
67	SF8_physicalhealth	Physical health summary score

Continued on next page

No	Feature	Description
68	SF8_sf01	"Overall, how would you rate your health during the past week?"
69	SF8_sf02	"During the past 4 weeks, how much did physical health problems limit your physical activities (such as walking or climbing stairs)?"
70	SF8_sf03	"During the past 4 weeks, how much difficulty did you have doing your daily work, both at home and away from home, because of your physical health?"
71	SF8_sf04	"How much bodily pain have you had during the past 4 weeks?"
72	SF8_sf05	"During the past 4 weeks, how much energy did you have?"
73	SF8_sf06	"During the past 4 weeks, how much did your physical health or emotional problems limit your usual social activities with family or friends?"
74	SF8_sf07	"During the past 4 weeks, how much have you been bothered by emotional problems (...) ?"
75	SF8_sf08	"During the past 4 weeks, how much did personal or emotional problems keep you from doing your usual work, school or other daily activities?"
76	SF8_social	Social functioning score
77	SF8_timestamp	SF8: avg. time needed to fill an item (in secs)
78	SF8_vitality	Vitality score
79	SOZK_abitur	Graduation: "Abitur"
80	SOZK_civservant	Occupation: civil servant
81	SOZK_divorced	Divorced
82	SOZK_employee	Occupation: employee
83	SOZK_fachabitur	Graduation: "Fachabitur"
84	SOZK_gender	Male gender
85	SOZK_geselle	Graduation: "Geselle"
86	SOZK_graduate	Graduation: university
87	SOZK_ill	"How long have you been ill during the last 12 months? (in months)"
88	SOZK_job	Job status: currently employed
89	SOZK_lowersec	Graduation: lower secondary school
90	SOZK_married	Married
91	SOZK_meister	Graduation: "Meister"
92	SOZK_mittlreife	Graduation: "mittlere Reife"
93	SOZK_nationality	German nationality
94	SOZK_nograd	Graduation: none
95	SOZK_nophysicians	"How many physicians have you visited because of your current complaints?"
96	SOZK_occupother	Occupation: other
97	SOZK_partnership	In partnership
98	SOZK_psychotreat	"How long have you been in psychotherapeutic treatment? (in months)"
99	SOZK_pupil	Graduation: currently pupil or "Azubi"
100	SOZK_selfempl	Occupation: self-employed
101	SOZK_student	Graduation: currently student
102	SOZK_timestamp	SOZK: avg. time needed to fill an item (in secs)
103	SOZK_tinnitusdur	"How long have you been suffering from tinnitus (in years)?"
104	SOZK_unemp	Duration of unemployment in the last 5 years
105	SOZK_unmarried	Unmarried
106	SOZK_worker	Occupation: worker
107	TESTING_day	testing date: day of the month

Continued on next page

No	Feature	Description
108	TESTING_month	testing date: month
109	TESTING_week	testing date: calendar week
110	TESTING_weekday	testing date: day of the week
111	TESTING_yearday	testing date: day of the year
112	TINSKAL_frequency	Tinnitus frequency
113	TINSKAL_impairment	Degree of tinnitus impairment
114	TINSKAL_loudness	Tinnitus loudness
115	TINSKAL_timestamp	TINSKAL: avg. time needed to fill an item (in secs)
116	TLQ_bothears	The tinnitus is located in both ears.
117	TLQ_entirehead	The tinnitus is located in the entire head.
118	TLQ_hissing	Tinnitus noise: hissing
119	TLQ_leftear	The tinnitus is located in the left ear.
120	TLQ_rightear	The tinnitus is located in the right ear.
121	TLQ_ringing	Tinnitus noise: ringing
122	TLQ_rustling	Tinnitus noise: rustling
123	TLQ_timestamp	TLQ: avg. time needed to fill an item (in secs)
124	TLQ_whistling	Tinnitus noise: whistling
125	TQ_audpercdiff	Auditory perceptual difficulties score
126	TQ_cogdistress	Cognitive distress score
127	TQ_distress	Total tinnitus distress score
128	TQ_emodistress	Emotional distress score
129	TQ_intrusivness	Intrusiveness score
130	TQ_psychodistress	Psychological distress score
131	TQ_sleepdisturb	Sleep disturbances score
132	TQ_somaticcompl	Somatic complaints score
133	TQ_timestamp	TQ: avg. time needed to fill an item (in secs)
134	TQ_tin01	I can sometimes ignore the noises even when they are there.
135	TQ_tin02	I am unable to enjoy listening to music because of the noises.
136	TQ_tin03	It's unfair that I have to suffer with my noises.
137	TQ_tin04	I wake up more in the night because of my noises.
138	TQ_tin05	I am aware of the noises from the moment I get up to the moment I sleep.
139	TQ_tin06	Your attitude to the noise makes no difference to how it affects you.
140	TQ_tin07	Most of the time the noises are fairly quiet.
141	TQ_tin08	I worry that the noises will give me a nervous breakdown.
142	TQ_tin09	Because of the noises I have difficulty in telling where sounds are coming from.
143	TQ_tin10	The way the noises sound is really unpleasant.
144	TQ_tin11	I feel I can never get away from the noises.
145	TQ_tin12	Because of the noises I wake up earlier in the morning.
146	TQ_tin13	I worry whether I will be able to put up with this problem for ever.
147	TQ_tin14	Because of the noises it is more difficult to listen to several people at once.
148	TQ_tin15	The noises are loud most of the time.
149	TQ_tin16	Because of the noises I worry that there is something seriously wrong with my body.
150	TQ_tin17	If the noises continue my life will not be worth living.
151	TQ_tin18	I have lost some of my confidence because of the noises.
152	TQ_tin19	I wish someone understood what this problem is like.

Continued on next page

No	Feature	Description
153	TQ_tin20	The noises distract me whatever I am doing.
154	TQ_tin21	There is very little one can do to cope with the noises.
155	TQ_tin22	The noises sometimes give me a pain in the ear or head.
156	TQ_tin23	When I feel low and pessimistic the noise seems worse.
157	TQ_tin24	I am more irritable with my family and friends because of the noises.
158	TQ_tin25	Because of the noises I have tension in the muscles of my head and neck.
159	TQ_tin26	Because of the noises other people's voices sound distorted to me.
160	TQ_tin27	It will be dreadful if these noises never go away.
161	TQ_tin28	I worry that the noises might damage my physical health.
162	TQ_tin29	The noise seems to go right through my head.
163	TQ_tin30	Almost all my problems are caused by these noises.
164	TQ_tin31	Sleep is my main problem.
165	TQ_tin32	It's the way you think about the noise - NOT the noise itself which makes you upset.
166	TQ_tin33	I have more difficulty following a conversation because of the noises.
167	TQ_tin34	I find it harder to relax because of the noises.
168	TQ_tin35	My noises are often so bad that I cannot ignore them.
169	TQ_tin36	It takes me longer to get to sleep because of the noises.
170	TQ_tin37	I sometimes get very angry when I think about having the noises.
171	TQ_tin38	I find it harder to use the telephone because of the noises.
172	TQ_tin39	I am more liable to feel low because of the noises.
173	TQ_tin40	I am able to forget about the noises when I am doing something interesting.
174	TQ_tin41	Because of the noises life seems to be getting on top of me.
175	TQ_tin42	I have always been sensitive about trouble with my ears.
176	TQ_tin43	I often think about whether the noises will ever go away.
177	TQ_tin44	I can imagine coping with the noises.
178	TQ_tin45	The noises never 'let up'.
179	TQ_tin46	A stronger person might be better at accepting this problem.
180	TQ_tin47	I am a victim of my noises.
181	TQ_tin48	The noises have affected my concentration.
182	TQ_tin49	The noises are one of those problems in life you have to live with.
183	TQ_tin50	Because of the noises I am unable to enjoy the radio or television.
184	TQ_tin51	The noises sometimes produce a bad headache.
185	TQ_tin52	I have always been a light sleeper.

Table 1: **Feature overview.** Overview of all 185 features which were used for classification. TQ: German version of the Tinnitus Questionnaire [5]; PSQ: Perceived Stress Questionnaire [3]; SF8: Short Form 8 Health Survey [4]; ADSL: General Depression Scale - long form [2]; SOZK: sociodemographics questionnaire; TINSKAL: visual analogue scales measuring tinnitus loudness, frequency and distress, TLQ: Tinnitus Localisation and Quality [6].

Supplementary B: hyperparameter tuning

In this study, 11 classification algorithms were employed: LASSO [8] (`lasso`), RIDGE (`ridge`), weighted k-nearest neighbour classifier [9] (`wknn`), Naïve Bayes classifier (`nb`), support vector machine [10] (`svm`), a feed-forward neural network with one single hidden layer [11] (`nnet`), generalised partial least squares [12] (`gpls`), CART decision tree [13] (`cart`), C5.0 decision tree [14] (`c5.0`), random forest [15] (`rf`) and gradient boosted trees [16] (`gbt`).

All classifiers were implemented with the statistical programming language R [17] using the package mlr[18], which provides a consistent interface to many machine learning algorithms from other R packages. A grid search was employed for hyperparameter tuning using area under the ROC curve (AUC) as evaluation measure. Table 2 provides an overview about each classifier, including used R package, tuned hyperparameters and their value ranges. All other hyperparameters were set to default values.

Algorithm (R package)	Hyperparameter	Min	Max	#Values
`lasso`, `ridge` (both `glmnet` [19])	`lambda`	0.01	10^{10}	100
`wknn` (`kknn` [9])	`k`	1	77	20
`svm` (`e1071` [20])	`cost`	0.01	3	6
	`gamma`	0	3	4
	`kernel`	*	-	4
`nb` (`e1071` [20])	`laplace`	1	5	5
`nnet` (`nnet` [21])	`size`	1	13	7
	`decay`	10^{-4}	1	6
`gpls` (`caret` [22])	`ncomp`	1	5	5
`cart` (`rpart` [23])	`cp`	0.001	0.1	5
`c5.0` (`C50` [24])	`CF`	0	0.35	7
	`rules`	`FALSE`	`TRUE`	2
	`winnow`	`FALSE`	`TRUE`	2
`rf` (`ranger` [25])	`mtry`	4	100	7
	`min.node.size`	1	25	6
`gbt` (`xgboost` [26])	`eta`	0.01	0.4	4
	`max_depth`	1	3	3
	`colsample_bytree`	0.2	1	5
	`min_child_weight`	0.5	2	3
	`subsample`	0.2	1	3
	`nrounds`	50	250	3

Table 2: **Value grid for hyperparameter tuning.** $^* = \{linear, polynomial, radial, sigmoid\}$

Supplementary C: best model

The classifier that induced the best model was `lasso` (AUC=0.87±0.04). For each feature selection iteration, Figure 1 shows a heatmap of model reliance (MR) scores for all features. After iteration $i = 1$, 89 out of the 185 features were kept for model training in $i = 2$. The feature selection wrapper converges at i7 with a model that uses 6 features, each with a MR exceeding 1. Notably, the removal of 97% of features from $i = 1$ to $i = 7$ let to much simpler model, while only dropping marginally in AUC (-0.02).

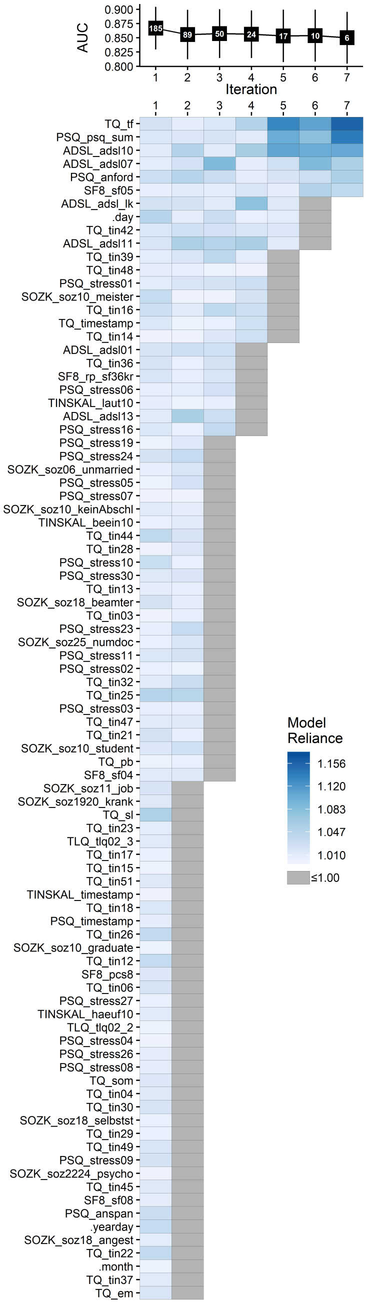

Figure 1: MR **convergence for `lasso` classifier.** Heatmap of retained and discarded features for each feature selection iteration for the `lasso` classifier. A blue-shaded cell represents a feature with a model reliance exceeding 1 whereas a grey cell represents a feature that was discarded from model training in the next iteration. The top subfigure shows AUC for the models of each iteration.

References

[1] Radloff, L. S. The CES-D scale: a self-report depression scale for research in the general population. *Applied psychological measurement* **1**, 385–401 (1977).

[2] Hautzinger, M. & Bailer, M. ADS-Allgemeine Depressionsskala. In *Diagnostische Verfahren in der Psychotherapie* (Beltz, 2003).

[3] Fliege, H. *et al.* The Perceived Stress Questionnaire (PSQ) reconsidered: validation and reference values from different clinical and healthy adult samples. *Psychosomatic medicine* **67**, 78–88 (2005).

[4] Bullinger, M. & Morfeld, M. Der SF-36 Health Survey. In *Gesundheitsökonomische Evaluationen*, 387–402 (Springer, 2008).

[5] Goebel, G. & Hiller, W. *Tinnitus-Fragebogen:(TF); ein Instrument zur Erfassung von Belastung und Schweregrad bei Tinnitus; Handanweisung* (hogrefe, Verlag für Psychologie, 1998).

[6] Goebel, G. & Hiller, W. Psychische Beschwerden bei chronischem Tinnitus: Erprobung und Evaluation des Tinnitus-Fragebogens (TF). *Verhaltenstherapie* **2**, 13–22 (1992).

[7] Brüggemann, P. *et al.* Impact of multiple factors on the degree of tinnitus distress. *Frontiers in human neuroscience* **10**, 341 (2016).

[8] Tibshirani, R. Regression shrinkage and selection via the lasso. *Journal of the Royal Statistical Society: Series B (Methodological)* **58**, 267–288 (1996).

[9] Hechenbichler, K. & Schliep, K. Weighted k-nearest-neighbor techniques and ordinal classification. In *SFB 386, Ludwig-Maximilians University, Munich*, vol. 399 of *sfb386* (2004). URL http://nbn-resolving.de/urn/resolver.pl?urn=nbn:de:bvb:19-epub-1769-9.

[10] Boser, B. E., Guyon, I. M. & Vapnik, V. N. A training algorithm for optimal margin classifiers. In *Proc. of Workshop on Computational Learning Theory*, 144–152 (ACM, 1992).

[11] Venables, W. N. & Ripley, B. D. *Modern Applied Statistics with S* (Springer, 2002), fourth edn.

[12] Ding, B. & Gentleman, R. Classification using generalized partial least squares. *Journal of Computational and Graphical Statistics* **14**, 280–298 (2005).

[13] Breiman, L., Friedman, J., Olshen, R. & Stone, C. *Classification and Regression Trees* (Wadsworth and Brooks, 1984).

[14] Quinlan, R. *C4.5: Programs for Machine Learning* (Morgan Kaufmann Publishers, San Mateo, CA, 1993).

[15] Breiman, L. Random forests. *Machine learning* **45**, 5–32 (2001).

[16] Friedman, J. H. Greedy function approximation: a gradient boosting machine. *Annals of Statistics* 1189–1232 (2001).

[17] R Core Team. *R: A Language and Environment for Statistical Computing*. R Foundation for Statistical Computing, Vienna, Austria (2018). URL https://www.r-project.org/.

[18] Bischl, B. *et al.* mlr: Machine Learning in R. *The Journal of Machine Learning Research* **17**, 5938–5942 (2016).

[19] Friedman, J., Hastie, T. & Tibshirani, R. Regularization paths for generalized linear models via coordinate descent. *Journal of Statistical Software* **33**, 1–22 (2010). URL http://www.jstatsoft.org/v33/i01/.

[20] Dimitriadou, E., Hornik, K., Leisch, F., Meyer, D. & Weingessel, A. e1071: Misc Functions of the Department of Statistics (e1071), TU Wien. R package version 1.5-25. (2011). URL http://cran.r-project.org/package=e1071.

[21] Venables, W. N. & Ripley, B. D. *Modern Applied Statistics with S* (Springer, New York, 2002), fourth edn. URL `http://www.stats.ox.ac.uk/pub/MASS4`. ISBN 0-387-95457-0.

[22] Kuhn, M. Building Predictive Models in R Using the caret Package. *Journal of Statistical Software, Articles* **28**, 1–26 (2008). URL `https://www.jstatsoft.org/v028/i05`.

[23] Therneau, T. & Atkinson, B. *rpart: Recursive Partitioning and Regression Trees* (2018). URL `https://CRAN.R-project.org/package=rpart`. R package version 4.1-13.

[24] Kuhn, M. & Quinlan, R. *C50: C5.0 Decision Trees and Rule-Based Models* (2018). URL `https://CRAN.R-project.org/package=C50`. R package version 0.1.2.

[25] Wright, M. N. & Ziegler, A. ranger: A fast implementation of random forests for high dimensional data in C++ and R. *Journal of Statistical Software* **77**, 1–17 (2017).

[26] Chen, T. & Guestrin, C. XGBoost: A Scalable Tree Boosting System. In *Proc. of ACM SIGKDD International Conference on Knowledge Discovery and Data Mining*, 785–794 (ACM, New York, NY, USA, 2016). URL `http://doi.acm.org/10.1145/2939672.2939785`.
